# Supplementary material for: The Effects of Local Weed Species on Arbuscular Mycorrhizal Fungal Communities in an Organic Winter Wheat (Triticum durum L.) Field in Lebanon
Source: Microorganisms. 2023 Dec 30;12(1):75. doi: 10.3390/microorganisms12010075 (PMC10819832; doi:10.3390/microorganisms12010075)
Supplement: Supplementary file 1 [file microorganisms-12-00075-s001.zip › microorganisms-2790090-supplementary.pdf]

SUPPLEMENTARY MATERIALS

Figure S1. AM fungal colonization in *T. durum* and weeds root samples (Td: *T. durum* without neighboring weeds; Td_w: *T. durum* surrounded by weeds; W_{Td}: weeds associated to *T. durum*; W: weeds on the edge outside the field). Data are mean ± SE (n=5). No significant differences were observed between samples (Parametric data, ANOVA; $P < 0.05$).

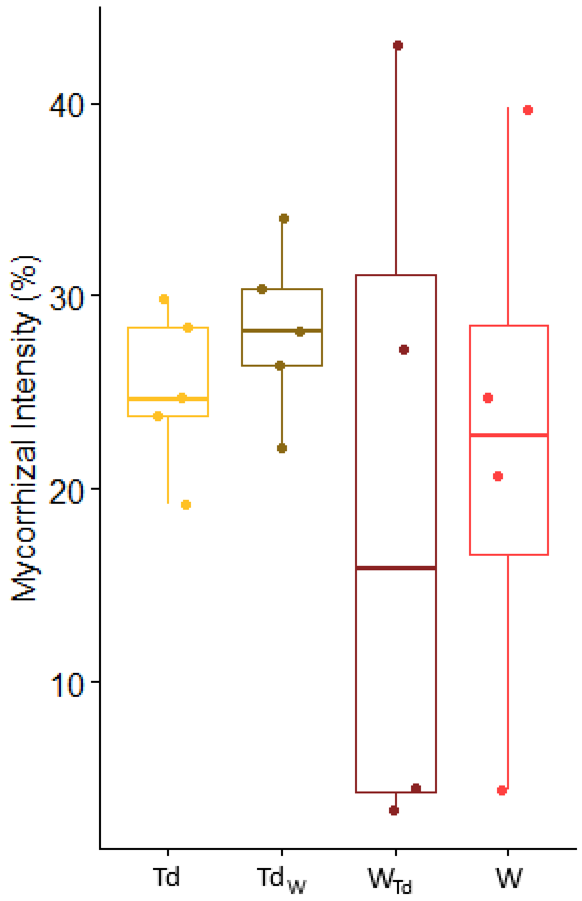

Figure S2. Rarefaction curves from the number of sequences in *T. durum* and weeds root samples (W: weeds on the edge outside the field (**a**); Td: *T. durum* without neighboring weeds (**b**) ; Td_{w}: *T. durum* surrounded by weeds (**c**); W_{Td}: weeds associated to *T. durum* (**d**))

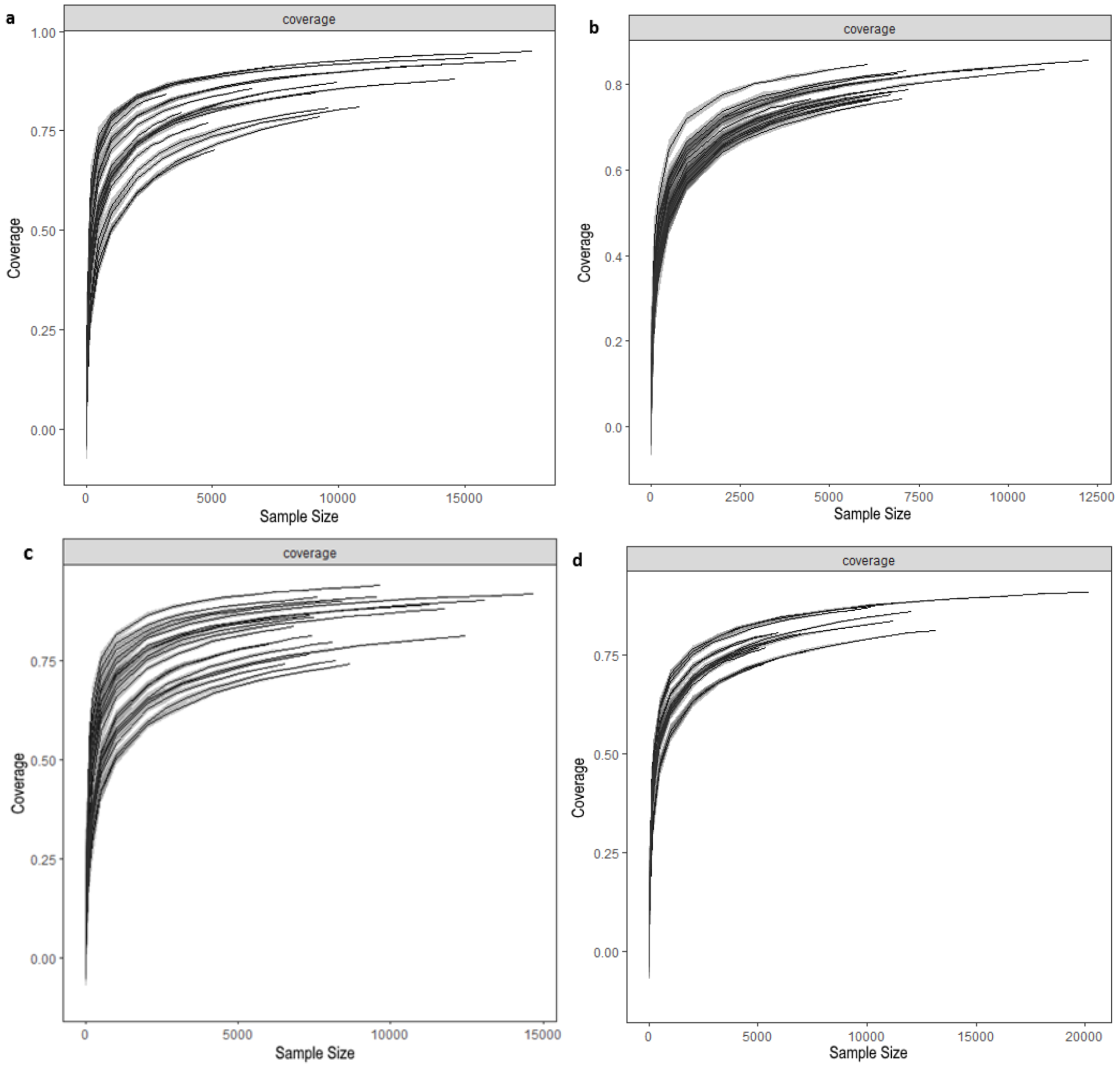

Figure S3. Richness (**a**) and Pielou's Evenness (**b**) of AMF community in *T. durum* and weeds root samples (Td: *T. durum* without neighboring weeds; Td_w: *T. durum* surrounded by weeds; W_{Td}: weeds associated to *T. durum*; W: weeds on the edge outside the field). No significant differences were observed between samples (Non-parametric data, Shapiro–Wilk test; P value < 0.05).

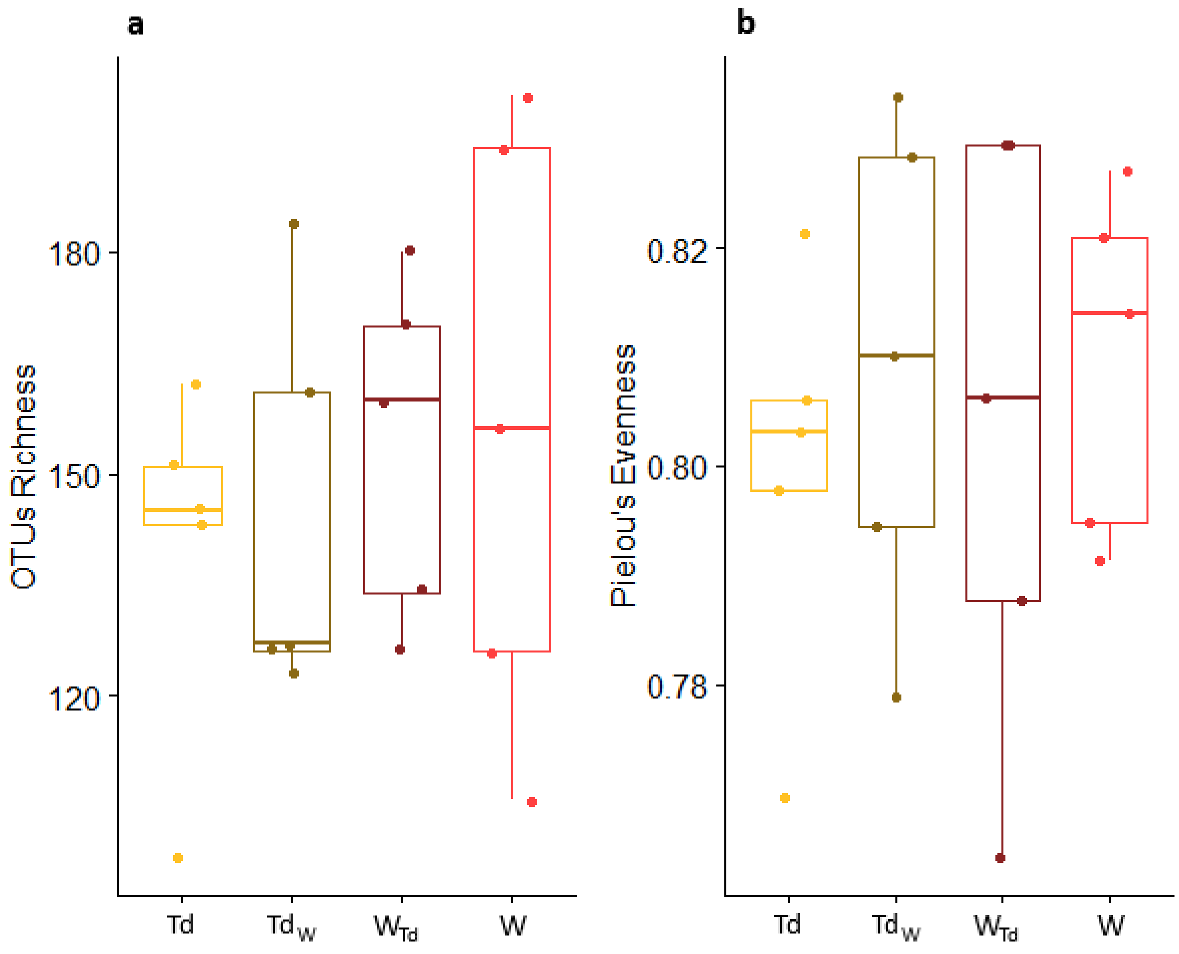

Figure S4. Effect of soil samples (Td_{soil}: soil from block samples covered with *T. durum* without neighboring weeds; TdW_{soil}: soil from block samples covered with *T. durum* surrounded with weeds) on shoot biomass (**a**) and root AM fungal colonization (**b**) in *Medicago sativa* in greenhouse. The asterisk indicates significant differences between samples (**a**: Normality of data not verified (Shapiro-Wilk test), means compared with Kruskal-Wallis test: P = 0.01613; **b**: data is parametric, means compared with ANOVA: P = 0.00107).

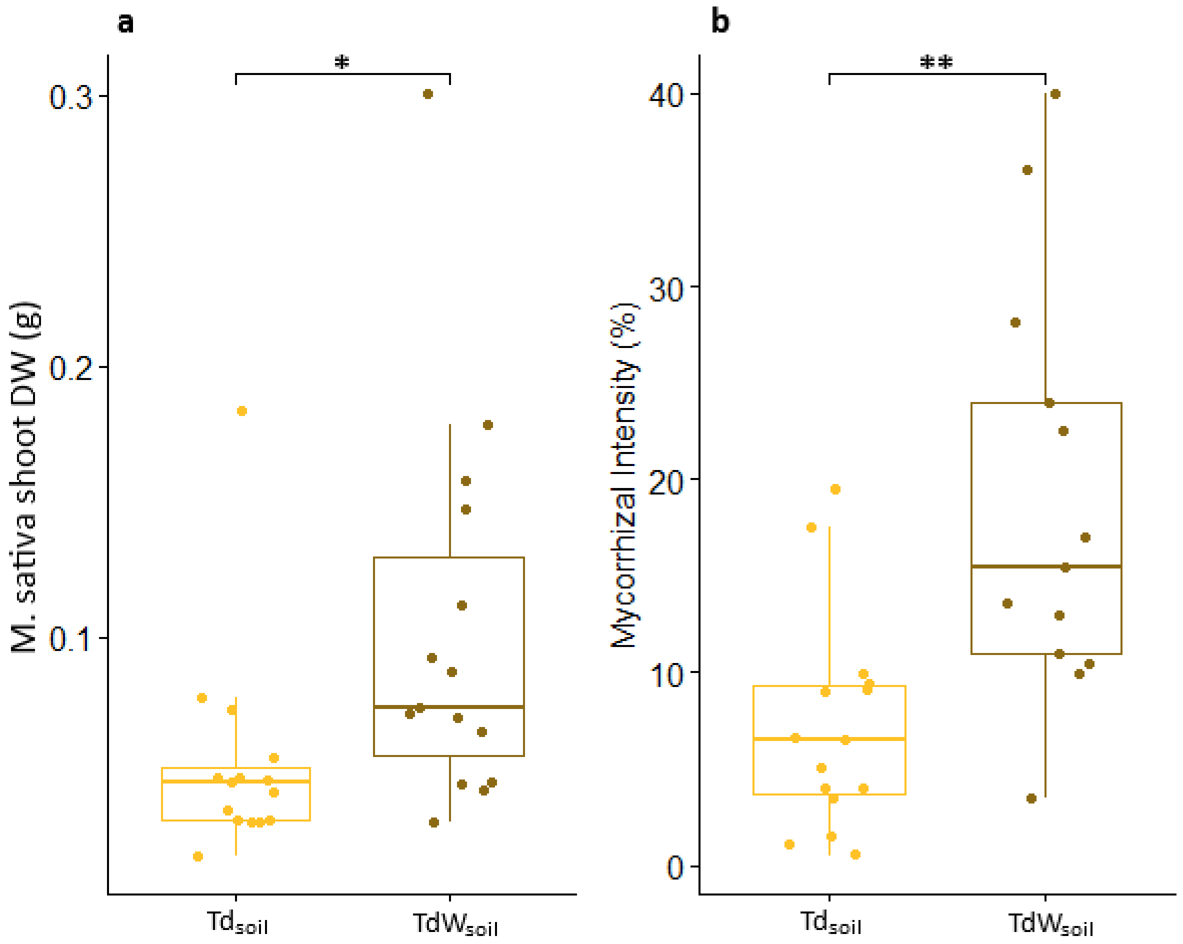

Table S1. Floristic inventory. Floristic states were performed in 0.5m x 0.5m quadrat at each location of the collected soil block samples (W: weeds on the edge outside the field; W_{Td}: weeds associated to *T. durum*).

Weed species	W samples					W_{Td} samples				
	Block 1	Block 2	Block 3	Block 4	Block 5	Block 1	Block 2	Block 3	Block 4	Block 5
Aegilops peregrina (Hack.) Maire & Weiller		x								
Ajuga chamaepitys subsp. chia (Schreb.) Arcang.	x									
Alopecurus utriculatus Sol., 1794	x		x							
Anisantha sterilis (L.) Nevski, 1934				x						
Anthemis chia L.										x
Avena sterilis L., 1762	x		x	x	x					
Biserrula pelecinus L., 1753					x					
Bromus scoparius L., 1755				x						
Calendula arvensis L., 1763			x				x	x	x	
Carduus argentatus L.		x	x	x	x					
Carlina curetum Halácsy			x							
Centaurea hyalolepis Boiss., 1846		x								
Crepis aspera L.	x		x					x		x
Echium judaeum Lacaita										x
Erodium malacoides (L.) L'Hér., 1789										x
Geropogon hybridus (L.) Sch.Bip., 1844	x			x						
Hirschfeldia incana (L.) Lagr.-Foss., 1847				x	x			x		x
Hordeum bulbosum subsp. bulbosum L., 1756			x							
Lamiaceae sp						x				
Linum pubescens Banks & Sol.	x		x	x						
Lomelosia prolifera (L.) Greuter & Burdet		x								
Lotus peregrinus L.	x	x		x	x					
Lysimachia arvensis (L.) U.Manns & Anderb., 2009	x	x	x							
Medicago rugosa Desr., 1792				x	x					
Mercurialis annua L., 1753			x		x		x	x		x
Muscari sp.					x					x
Notobasis syriaca (L.) Cass., 1825		x			x					
Picris galilaea (Boiss.) Eig	x	x	x		x					x
Pimpinella cretica Poir.		x								
*Plantago afr*a L., 1762								x		
Reichardia intermedia (Sch. Bip.) Samp.		x								
Rhagadiolus stellatus (L.) Gaertn., 1791	x		x			x	x	x	x	x
Scandix pecten-veneris subsp. pecten-veneris L., 1753	x	x	x	x	x					
Theligonum cynocrambe L., 1753	x			x	x		x		x	
Tordylium syriacum L.		x							x	
Torilis arvensis (Huds.) Link, 1821		x								
Torilis tenella (Delile) Rchb.f., 1867	x			x						
Trifolium clusii Godr. & Gren., 1849		x								
Trifolium stellatum L., 1753			x							

Table S2 . Chemical properties of soil samples (Td_{soil}: soil from block samples covered with *T. durum* without neighboring weed plants; TdW_{soil}: soil from block samples covered with *T. durum* surrounded with weeds; W_{soil}: soil from block samples covered with weeds on the edge outside the wheat field). Data are mean ± SE (n=5). Different letters following the same column indicate a significant difference between soils (Parametric data, means compared with ANOVA; $P < 0.05$).

Study area	Sampled soil	pH	Total N (%)	Total C (%)
Inside the field	TdW_{soil}	8.05 ± 0.05 [a]	0.31 ± 0.05 [a]	4.53 ± 0.43 [a]
	Td_{soil}	8.09 ± 0.09 [a]	0.21 ± 0.02 [b]	3.45 ± 0.26 [b]
In the border outside the field	W_{soil}	8.13 ± 0.09 [a]	0.25 ± 0.05 [ab]	4.24 ± 0.81 [ab]
